# The Epidemiology of Violent Deaths in Chile between 2001 and 2018: Prevalence, Trends, and Correlates

**DOI:** 10.3390/ijerph191912791

**Published:** 2022-10-06

**Authors:** Katjana Wiederkehr, Caroline Mai, José M. Cabezas, Teresita Rocha-Jiménez, Tamara Otzen, Nicolás Montalva, Esteban Calvo, Alvaro Castillo-Carniglia

**Affiliations:** 1 Mailman School of Public Health, Columbia University, New York, NY 10032, USA; 2 Society and Health Research Center, Facultad de Ciencias Sociales y Artes, Universidad Mayor, Santiago 7560908, Chile; 3 Millennium Nucleus for the Evaluation and Analysis of Drug Policies (nDP), Santiago 7560908, Chile; 4 Millennium Nucleus on Sociomedicine (SocioMed), Santiago 7560908, Chile; 5 Center of Morphological and Surgical Studies (CEMyQ), Ph.D. Program in Medical Sciences, Universidad de la Frontera, Temuco 4811230, Chile; 6 CalvoLab, Laboratory on Aging and Social Epidemiology, Santiago 7560908, Chile

**Keywords:** mortality, violence, homicide, suicide, Chile

## Abstract

Background: Despite its enormous health and social burden, there are limited published studies describing the epidemiology of violent deaths in Chile. We described violent mortality rate trends in Chile between 2001 and 2018, its current spatial distribution and ecological level correlates. Methods: A population-based study using publicly accessible data. We calculated age-adjusted mortality rates per 100,000 persons for sex, age, intention, and mechanism of death. Next, we used linear regression to estimate time trends for sex and intention. We then employed hierarchical Poisson analyses to model the spatial distribution across 345 municipalities and the influence of six ecological level variables. Results: The average rate of violent death in Chile between 2001 and 2018 was 15.9 per 100,000 people, with the majority (70.3%) of these attributed to suicide. Suffocation was the most common mechanism of death for suicide (82.3%) and cut/pierce for homicide (43.1%), followed by firearm (33.2%). Violent deaths are trending downward in Chile across all categories except suicides by women, which have remained stable. Poverty rates and urban population were positively associated with violent mortality rates. Conclusions: Although violence-related deaths seem to be decreasing, disparities across gender, age group, and geographic location may have continuing effects on mortality rates.

## 1. Introduction

The impact of violence on global public health has been recognized as an important concern for mortality rates throughout the world [1]. It is the fourth leading cause of death for individuals aged 15–44 years old and accounts for more than 1.3 million deaths each year (2.5% of all global deaths) [1]. As defined by the World Health Organization, violence is “the intentional use of physical force or power, threatened or actual, against oneself, another person, or against a group or community, that either results in or has a high likelihood of resulting in injury, death, psychological harm, maldevelopment, or deprivation,” [2]. Deaths resulting from violence also influence life expectancy, socio-economic development, mental and physical capabilities, and high-risk behavior in affected communities [1].

Violence is caused by many social, environmental, and economic factors, and it is fostered by both individual- and community-based ideology and action. Evidence shows that unemployment, gender inequality, income inequality, and residential instability are all significantly associated with homicide and suicide rates [3]. Due to the cultural and social variability of these determinants as well as preventative measures, studies have also shown high heterogeneity across interpersonal violence [4]. Inequality and heterogeneity lead to intrinsic internal and external relationships and tensions that parallel social, economic, and geographic power structures [5]. Struggles within these relationships and structures have the potential to manifest in violent actions. 

Latin America has faced an increase in social and interpersonal violence over the past few years. Much of this increase can be attributed to political transitions and the multifaceted influences of socioeconomic status and cultural behavior [6]. As of 2017, Latin America has one of the highest rates of homicide in the world at 24.7 per 100,000 persons and is home to 42 of the 50 most violent cities in the world [7]. When looking at suicide, the rates in Latin America are lower compared to the rest of the world at 5.2 per 100,000 persons; however, this number is more indicative of low-income countries and is trending upwards [8].

These results, however, are far from being equal across Latin American countries and even within countries with smaller administrative areas. For example, Chile exhibits lower rates of homicide than the rest of Latin America, with a rate of 3.9 per 100,000 people in 2012 [9]. On the other hand, for suicides, the country has some of the highest numbers in the region with a rate of 12.6 per 100,000 people in 2011, similar to the European Union and other high-income countries [10]. Chile has been recently classified as a developing high-income country with a growth domestic product per capita of about USD 16,000 and a low unemployment rate (7.2% before the COVID-19 pandemic). It has an annual growth domestic product of USD 253 billion and an emergent economy based on mining, agriculture, and a growing tertiary economic activity [11]. Although Chile has made great economic gains in the past few years, it still has a large amount of income inequality [12]. It has a population of about 19 million, mostly aggregated in urban centers (88%) and in the Metropolitan Region (41%) where its capital city Santiago is [12]. In addition to income equality, high rates of suicide could negatively impact Chile’s economic growth in the form of lost productive years of life. Studies have shown a positive correlation between gross domestic product growth and suicide rates in Latin America, and this effect could be why Chile’s suicide rates have increased in parallel to its economy [13].

To our knowledge, the most recent articles on this topic only include data up until 2011 for suicide [10,14] and 2012 for homicides [9]. Additionally, within-country variation in Chile is largely unknown, though comparative evidence and non-academic publication show that violent death tends to be concentrated in more populated areas, particularly homicides [6,9,15].

The objective of our study was to describe violent mortality rate trends in Chile between 2001 and 2018, by intention (i.e., homicide, suicide) and mechanism (e.g., firearms, poisoning), and it included a spatial analysis to model homicides and suicides for the smallest administrative area available in Chile (municipalities). Knowing the evolution and distribution of violent mortality in Chile, as well as the explanatory power of municipal-level variables, we can inform decision makers and contribute to the generation of policies to prevent and control violence at the national and local levels.

## 2. Materials and Methods

### 2.1. Data and Design

The present investigation is a population-based study of people with a violent cause of death nationwide between 2001 and 2018. We utilized publicly accessible databases (https://deis.minsal.cl/#datosabiertos, accessed on 8 November 2021) from the Department of Information and Health Statistics from the Chilean Ministry of Health [16], which contain basic individual-level demographic information (e.g., sex, age, municipality of residency) of each deceased person, as well as the underlying and external (if any) cause of death.

### 2.2. Variables

The variables included are year, age, sex, and municipality, as well as intention and method of death. First, data were filtered based on people with an intentional violent external cause of death. This was performed by establishing two main categories of deaths related to violence: homicide and suicide. We excluded unspecified intentions (15.4% of total external causes of deaths in the period 2001–2018) since there was no method to determine whether these deaths were truly intentional or accidental and including them would affect the study’s assumptions and results.

We identified deaths using codes from the International Classification of Diseases 10th revision [17]. Homicide was defined as “injuries inflicted by another person with intent to injure or kill, by any means,” and included codes X85-X99, Y00-Y09, and Y87.1 [17,18]. Suicide was defined as “intentionally self-inflicted injury that results in death,” and included codes X60-X84 and X87.0 [18,19]. During the period between 2001 and 2018, Chile was not in a state of war or political unrest; therefore, we decided not to include violent deaths resulting from legal intervention and war operations. The overall violent deaths between these two categories were also explored. We then further coded these variables of intent by the method of violence: cutting/piercing, drowning, falling, fire/heat damage, firearm, vehicular, poisoning, striking, suffocation, and undetermined methods. Specific ICD-10 codes used for each of these methods are presented in Appendix A Table A1.

To be consistent with prior reports [10,19], we divided age into five categories (0–14, 15–24, 25–49, 50–64, and 65+). The spatial component of the study considered the 345 residential municipalities in Chile.

We also extracted ecological level variables from the National Municipal-level Information System to explore and explain potential sources of heterogeneity at the municipal level. The variables included cover four domains pointed out in prior studies as relevant dimensions of violence [20,21]: demographic (population density per square kilometer, percentage of the municipal population that is urban), socio-economic (percentage of municipal population living under the poverty line and school attendance rate), built environment (maintained green areas in square meters per inhabitant), and security (federally funded security staff per 100,000 people).

### 2.3. Data Analysis

We calculated the crude and age-adjusted mortality rates per 100,000 people for total violent deaths, intention, and method. The age-adjusted rates were calculated through the direct method using the 2018 Chilean population residents as the standard population. We reported rates across the years 2001–2018 and calculated time trends by means of simple linear regression using year as the independent variable and categorizing by gender and intention.

In addition, we estimated the standardized mortality ratio (SMR) for each municipality and each category of intent. To obtain the SMR $(SMR_{i}=O_{i}/E_{i}$), we estimated observed deaths ($O_{i})$ and then estimated the expected number of deaths ($E_{i})\;$through the indirect method, using national population estimates as provided by the National Institute of Statistics of Chile [22].

Since mortality estimates can be imprecise with a high degree of variability in small geographic areas or areas with low populations, we used Poisson hierarchical models to smooth the SMR at the municipal level for each intention and mechanism. This assumed that death counts follow a Poisson distribution, with death counts nested within municipalities. Smoothed municipal-level SMR were then generated through Empirical Bayes predictions, which combine prior information regarding violent deaths with the Poisson likelihood function. The extra Poisson variability was modeled by introducing a municipality-specific random intercept. To avoid a large proportion of zero count in at the municipal level, we aggregated deaths from the years 2016–2018. After smoothing, the SMRs were categorized into 5 groups using cutoff points previously used in mortality atlases [23]. After running intercept-only Poisson hierarchical models, we added covariates representing sociodemographic and security variables at the municipal level to determine any relationship between these characteristics and suicides or homicides. Pairwise correlation showed no evidence of collinearity between covariates (i.e., assuming a common threshold of r = 0.5–0.7), with poverty and urbanicity being the highest correlated variables (r = 0.451) [24]. Additionally, poverty can have different meanings across rural-urban settings. To test that hypothesis, in our data, we include interaction terms between urbanicity and poverty; however, in both suicide and homicide models, there was no evidence of interaction (*p* = 0.78 and *p* = 0.82 for the interaction term, respectively).

RStudio v. 1.4.1717 (Boston, MA, USA) statistical program was used for data management and cleaning, generating descriptive statistics, estimating observed and expected statistics on morality, and presenting the results in maps. Stata 16.0 (College Station, TX, USA) was used for Poisson modeling.

## 3. Results

**Figure 1:**
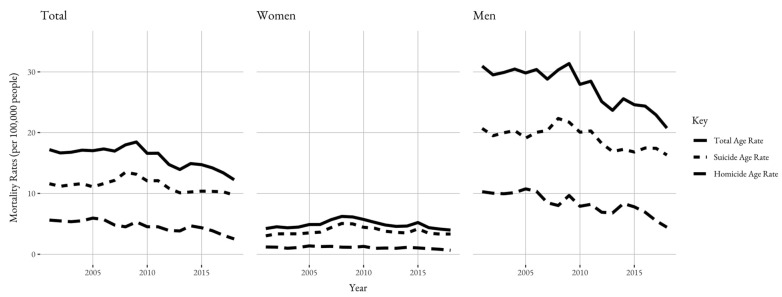
Age-adjusted violent mortality rates in Chile, 2001–2018.

Between 2001 and 2018, there were 47,113 violent deaths in Chile, with the majority of these attributed to suicide (33,124, 70.3%) (Table 1). The average age-adjusted mortality rate for this time was 15.9 per 100,000. Men accounted for the majority of violent deaths (39,650, 84.2%) and had higher mortality rates than women across each category for all years. The age group with the most violent deaths was people aged 25–49 (23,809, 50.5%). Suffocation was the most common method of death (27,651, 58.7%) overall and for suicides (27,254, 82.3%), while cutting/piercing was the most common method of death for homicides (6,029, 43.1%). In the overall population, there was significant evidence of a downwards trend in total violent deaths (*β* = −0.26; *p* < 0.001) (Table 2 and Figure 1). This is equivalent to a 1.7% annual decrease on average, or about 44 fewer deaths per year.

### 3.1. Suicide

There were 33,124 suicides in Chile between 2001 and 2018. A total of 27,304 (82.4%) of these deaths were men, and the mean age was 42 (Table 1). The most prominent methods of suicide were suffocation (82.3%), firearm (7.0%), and poisoning (5.6%). The average age-adjusted mortality rate in the period of 2016-2018 for suicides was 10.1 per 100,000 (Table 3). Suicide mortality rates were significantly higher for men than women, with the average rate for men at 17.1 per 100,000 and 3.4 per 100,000 for women between 2016 and 2018. Time trends reveal that suicide rates are trending downwards across the overall population with an average of 17 fewer deaths each year (*β* = −0.11; *p* = 0.019). Rates for men are also trending downwards (*β* = −0.24; *p* = 0.001). Suicides remain stable for women (*β* = 0.01; *p* = 0.761). However, suicide trends for women were not statistically significant (Table 2 and Figure 1).

### 3.2. Homicide

There were 13,989 homicides in Chile between 2001 and 2018. A total of 12,346 (88.3%) of these deaths were men, and the mean age was 35. The most prominent methods of homicide were cutting/piercing (43.1%), firearm (33.2%), and undetermined (17.7%). The average age-adjusted mortality rate in the period 2016–2018 for homicides was 3.2 per 100,000 (Table 3). Homicide mortality rates were significantly higher for men than women, with the average rate for men at 5.6 per 100,000 and 0.8 per 100,000 for women. Rates for both genders seem to be decreasing in magnitude and time trends show a downwards trend across the years in the overall population by 3.6% each year (*β* = −0.15; *p* < 0.001), as well as in women (*β* = −0.02; *p* = 0.003) and men (*β* = −0.30; *p* < 0.001) (Table 2 and Figure 1).

### 3.3. Spatial Analysis

**Figure 2:**
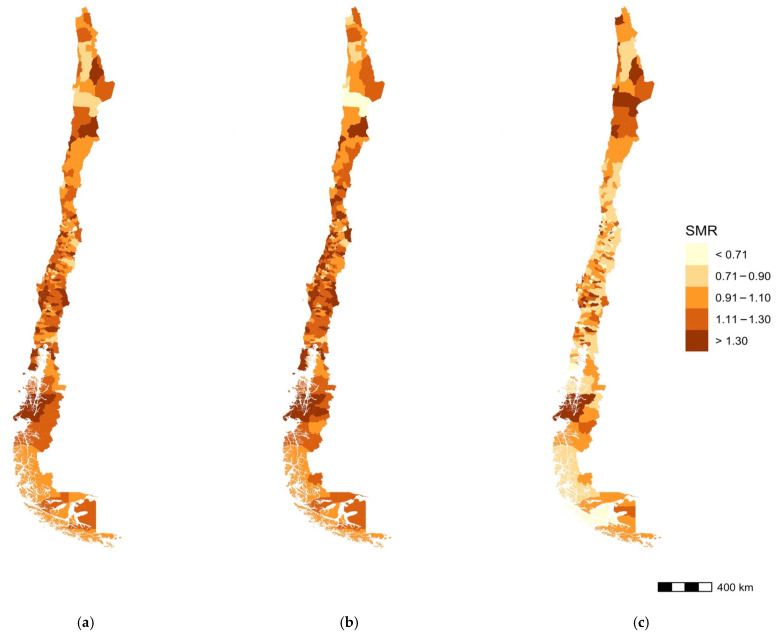
Smoothed Standardized Mortality Ratios (SMR) for (**a**) total deaths, (**b**) suicides and (**c**) homicides, 2016–2018.

Figure 2 displays the geographic distribution of smoothed SMRs for total deaths, suicides, and homicides between the years of 2016 and 2018. From the maps, we observed heterogeneity in total death, suicide, and homicide rates across municipalities. 

Overall, there was a concentration of municipalities with elevated risk (>30% of expected) of suicide in the south-central regions of Chile. The Araucanía Region had 13 (40%) municipalities with elevated risk, followed by Maule, Ñuble, and Biobio. For homicides, the regions with the largest proportion of municipalities with elevated risk were Arica y Parinacota (33%), followed by Antofagasta (22%), Araucanía (22%), and the Metropolitan Region (19%).

Furthermore, we modeled suicides and homicides using sociodemographic characteristics at the municipal level as explanatory variables. Table 4 shows that, while controlling for all other covariates, the only variable significantly associated was poverty; a 1% increase in poverty at the municipal level was associated with a 2% (95% CI: 1.01, 1.03) increase in suicide rates. For homicide, the percentage of urban population and poverty were significantly associated (Table 4); a 1% increase in the urban population at the municipal level was associated with a 1% (95% CI: 1.00, 1.02) increase in homicide rates and a 1% increase in poverty was associated with a 4% (95% CI: 1.03, 1.05) increase in homicide rates.

## 4. Discussion

This study provides a thorough description and analysis of violent deaths in Chile over the past two decades, considering the method of violence, its distribution across sex, age, and small geographical areas. The average rate of violent death in Chile between 2001 and 2018 was 15.9 per 100,000 people. Suicide was the most common violent death and rates were much higher among men than women. Ages 15–49 experienced the most violent deaths, which is consistent with global statistics [1]. Our findings show that overall, violent deaths have been decreasing in Chile over this period in a statistically significant way, with an average annual decrease of 1.7% for total violent deaths, 0.9% for suicide, and 3.6% for homicide.

Violent mortality rates in Chile between 2016 and 2018 for homicide at 3.2 per 100,000 people and suicide at 10.1 per 100,000 people are comparable to world mortality rates (homicides at 5.3 per 100,000 people and suicides at 9.2 per 100,000 people) but different from the standard in other Latin American countries in which homicide exceed suicides [7,25,26]. The gap between suicide and homicide rates seemed to widen significantly around 2005 and then remained steady with minor fluctuations. A stronger decrease in men’s homicides and a slight increase in women’s suicides may widen this gap further. When stratified by sex, total violent deaths and suicide rates showed stable trends over the past two decades for women, while for men they have significantly decreased. Although men’s suicide rates are higher than women’s, they are decreasing and so directing and increasing future efforts around the issue of suicides by women could help to impact these mortality rates. Recent policies to address inequities in mental health care access during the coronavirus pandemic could be utilized to expand on mental health foundations in Chile, and inform targeted interventions for women [27]. More research could also be conducted on potential associations between domestic violence and female suicide, and work to integrate methods of prevention.

The causal factors of violence are complex, and the trends seen in this study are unlikely to be the results of a singular phenomenon. Decades of socio-economic, cultural, and political change in Chile have surely impacted these trends in some way. It could be hypothesized that the decline in the homicide rate may be due to a political and social emphasis on security, increased police presence, decreased poverty, and improvements in education. Likewise, suicide rates may be decreasing due to an increased social awareness of mental health, and government policies and initiatives to prevent suicide and associated risk factors. It is possible that the differences between sexes seen in suicide trends might allude to initiatives’ ability to decrease already high male suicide rates, but they do not make enough of an impact to stop an increase in the suicide rates of females, who are often more likely to make repeated suicide attempts than men [28]. The Chilean government may also be more concerned with addressing other types of violence affecting women, such as domestic violence.

As shown by previous studies, our results also corroborate that violent mortality rates are heterogeneous across the country. From our models, which included socioeconomic indicators, we found that few variables were associated with these differences across municipalities. The poverty index was positively associated with suicides and homicides; the urban population was also positively associated with homicide rates. This suggests that creating and implementing initiatives that target structural social drivers, such as poverty, could be an effective approach to influencing violent mortality in Chile.

Limitations of the study are noted. There are issues with the amount and quality of data concerning violent deaths in Chile. A lack of consistent and accurate reporting limited our study to the years 2001 to 2018. The International Classification of Diseases 9th revision [29] was used in Chile before 1997, leading to concerns about the validity and quality of conversions between coding systems in research [30]. Furthermore, a high number of violent deaths were also coded as unspecified or undetermined before 2001, providing evidence for a skew in how certain deaths were being recorded and influencing the true number of homicides and suicides in the population. This may have been impacted by the switch from ICD-9 to ICD-10 and revision of the coding protocol. From 2019 to 2021, the public reporting of deaths does not include external causes of death and has been heavily affected by the magnitude of the coronavirus pandemic, creating gaps in Chile’s most current data on violent deaths.

Based on these findings, methods of intervention and prevention can be better informed to address the causes and effects of violent deaths throughout Chile. More research should be conducted on possible associations and correlating factors, as well as specific demographics. Moving forward, policy and intervention should focus on suicides, specifically for females and all persons aged 15–49. Expanding mental health access and awareness, potentially through social media campaigns, school programs, and increased services for low-income communities, may help further decrease suicides in Chile.

## 5. Conclusions

Across Chile, violent deaths such as homicides and suicides are trending downwards. While disparities in violent mortality rates between sexes and geographical areas remain, progress is being made to decrease violent deaths. Because trends of suicide by women have become relatively static, efforts to determine possible contributors of this issue could help to better understand women’s mental health risks and the effectiveness of suicide prevention initiatives by sex. Violent mortality rates by municipality show heterogeneity across the country. Structural variables such as poverty and urbanicity seem to have a role in shaping mortality distribution in Chile. These findings should help to inform and influence future initiatives in reducing and preventing violent deaths throughout Chile.

## Figures and Tables

**Table 1:** Violent death in Chile by intention, 2001–2018.

	Total	Suicide	Homicide
N (%) *	47,113 (100)	33,124 (70.30)	13,989 (29.70)
Gender			
Women	7463 (15.84)	5820 (17.57)	1643 (11.75)
Men	39,650 (84.16)	27,304 (82.43)	12,346 (88.25)
Age, y			
0–14	773 (1.64)	410 (1.24)	363 (2.59)
15–24	9729 (20.65)	5961 (18.00)	3768 (26.94)
25–49	23,809 (50.54)	16,289 (49.18)	7520 (53.76)
50–64	8151 (17.30)	6562 (19.81)	1589 (11.36)
65+	4651 (9.87)	3902 (11.78)	749 (5.35)
Method			
Cut/Pierce	6323 (13.42)	294 (0.89)	6029 (43.10)
Drowning	227 (0.48)	191 (0.58)	36 (0.26)
Fall	540 (1.15)	531 (1.60)	9 (0.06)
Fire/Heat	256 (0.54)	200 (0.60)	56 (0.40)
Firearm	6967 (14.79)	2318 (7.00)	4649 (33.23)
Vehicular	104 (0.22)	102 (0.31)	2 (0.01)
Poisoning	1851 (3.93)	1837 (5.55)	14 (0.10)
Struck	352 (0.74)	31 (0.09)	321 (2.29)
Suffocation	27,651 (58.69)	27,254 (82.28)	397 (2.84)
Undetermined	2842 (6.03)	366 (1.10)	2476 (17.70)

* Percentages reported are column percentages.

**Table 2:** Time trends for violent mortality rates by gender and intention, Chile, 2001–2018.

	*β*	95% CI	*p*
Overall			
Total	−0.26	(−0.36, −0.16)	<0.001
Suicide	−0.11	(−0.19, −0.02)	0.019
Homicide	−0.15	(−0.20, −0.11)	<0.001
Women			
Total	−0.01	(−0.08, 0.05)	0.668
Suicide	0.01	(−0.05, 0.07)	0.761
Homicide	−0.02	(−0.04, −0.01)	0.003
Men			
Total	−0.54	(−0.69, −0.39)	<0.001
Suicide	−0.24	(−0.37, −0.12)	0.001
Homicide	−0.30	(−0.38, −0.22)	<0.001

**Table 3:** Average age-adjusted mortality rates in Chile by sex, age, and method, 2016–2018.

	Total	Suicide	Homicide
2001–2003	16.87	11.39	5.48
2016–2018	13.27	10.10	3.17
Sex			
Women	4.16	3.36	0.80
Men	22.67	17.06	5.62
Age *			
0–14	0.10	0.01	0.20
15–24	1.77	1.07	0.52
25–49	13.33	12.17	4.50
50–64	1.71	1.06	0.17
65+	1.23	0.91	0.14
Method			
Cut/Pierce	1.39	0.10	1.29
Drowning	0.04	0.04	0.00
Fall	0.17	0.16	0.00
Fire/Heat	0.05	0.04	0.00
Firearm	1.70	0.41	1.30
Vehicular	0.04	0.04	0.00
Poisoning	0.34	0.34	0.00
Struck	0.07	0.00	0.07
Suffocation	8.99	8.90	0.09
Undetermined	0.49	0.08	0.41

* Rates by age are not age-adjusted.

**Table 4:** Municipal level correlated for suicide and homicides in Chile, 2016–2018.

	Suicide		Homicide	
	Rate Ratio	95% CI	Rate Ratio	95% CI
Population density (per square kilometer)	0.99	(0.99, 1.00)	1.00	(0.99, 1.00)
Urban population (%)	0.99	(0.99, 1.00)	1.01	(1.00, 1.02)
Poverty (%)	1.02	(1.01, 1.03)	1.04	(1.03, 1.05)
School attendance (%)	1.01	(0.99, 1.02)	0.99	(0.97, 1.02)
Maintained green areas (square meter per inhabitant)	0.99	(0.99, 1.01)	1.00	(0.98, 1.02)
Security staff (per 100,000 people)	0.99	(0.99, 1.00)	1.00	(0.99, 1.00)

## Data Availability

The datasets used during the current study are publicly available from public sources in the Department of Information and Health Statistics from the Chilean Ministry of Health (https://deis.minsal.cl/, accessed on 8 November 2021) and the National Municipal-level Information System (http://datos.sinim.gov.cl/datos_municipales.php, accessed on 8 November 2021).

## Appendix A

**Table A1:** ICD-10 Codes for violent deaths.

	Suicide	Homicide
Cut/Pierce	X78	X99
Drowning	X71	X92
Fall	X80	Y01
Fire/Heat	X76, X77	X97, X98
Firearm	X72–X74	X93, X94, X95
Motor vehicle	X82	Y03
Poisoning/exposure to chemicals and noxious substances	X60–X69	X85–X90
Struck	X79, X81	Y00, Y04
Threat to breathing	X70	X91
Other/undetermined	X75, X83, X84, Y87.0	X96, Y02, Y05–Y09, Y87.1
